# Semantic segmentation dataset of Land Use/Cover Area frame Survey (LUCAS) rural landscape Street View Images

**DOI:** 10.1016/j.dib.2024.110394

**Published:** 2024-04-06

**Authors:** Laura Martinez-Sanchez, Koen Hufkens, Elizabeth Kearsley, Dimitar Naydenov, Bálint Czúcz, Marijn van de Velde

**Affiliations:** a Joint Research Center, European Commission, Ispra, Italy; b BlueGreen Labs, Melsele, Belgium; c HD Solutions LTD, Sofia, Bulgaria; d Norwegian Institute for Nature Research, Trondheim, Norway

**Keywords:** Land-use and land-cover change, Segmentation, Biodiversity, Machine learning, Monitoring, Vector, Image, Street level imagery, Landscape elements, Computer vision

## Abstract

Urban focused semantically segmented datasets (e.g. ADE20k or CoCo) have been crucial in boosting research and applications in urban areas by providing rich sources of delineated objects in Street View Images (SVI). However, there is a lack of similar datasets for agricultural and rural landscapes. By focusing on such underrepresented landscapes, we created a dataset containing images with visually segmented objects that were labeled following a thematically relevant set of classes. The dataset contains 1784 north-looking landscape images with their corresponding annotated masks from across Europe. Images were sourced from the Land Use and Coverage Area frame Survey (LUCAS), following a strict sampling and acquisition protocol. Objects were fully delineated on the street (eye) level or so-called landscape images for a set of 35 relevant classes (e.g. cropped fields, dense woody features, field margins, stone walls). This modest dataset, due to the cost of segmentation, might provide limitations for some applications (due to class imbalances). However, initial segmentation labels open the potential for the rapid (semi-supervised) growth of a larger dataset using LUCAS or other street level imagery. Although uncertainties remain, this annotated dataset is a first step toward integrating LUCAS image data within a landscape segmentation context. This can support land-use and land-cover change assessments, comparison and integration with Earth Observation based products, improved structural characterization of vegetation, as well as biodiversity and landscape heterogeneity monitoring. The data are structured in two folders which store the images and the masks. Also included is a csv file with the label and codes corresponding to the masks and another csv file data with geolocation information and ancillary data derived from the Harmonized LUCAS in-situ land-cover and land-use database for each image.

Specifications Table

**Table:** 

Subject	Environmental Sciences (Management, Monitoring, Policy and Law)
Specific subject area	Street level image data for machine learning of land-cover / land-use and biodiversity monitoring
Data format	Raw, Analyzed, Filtered RAW: JPEG Annotations/masks: PNG
Type of data	Table, Image
Data collection	The data presented are segmentation masks of 35 classes generated using the CVAT software based on a subset of north looking landscape SVI of the LUCAS database of the 2018 survey. Images are resized to a consistent 1600 × 1200 pixels and in jpg format, segmentation masks are provided with the same resolution as PNG images. The dataset covers a total of 27 EU countries (AT, BE, BG, CY, CZ, DE, DK, EE, EL, ES, FI, FR, HR, HU, IE, IT, LT, LU, LV, NL, PL, PT, RO, SE, SI, SK, UK)
Data source location	https://data.jrc.ec.europa.eu/dataset/adace32a-465f-412b-bc11-be1bc06322d3 European Commission - Joint Research Centre VIA ENRICO FERMI 2749 - 21,027 Ispra (VA). Italy
Data accessibility	Repository name: Zenodo and source.coop Direct URL to data: https://beta.source.coop/repositories/jrc-lucas/jrc-lucas-ml/description/ Example us ML use of the data: bluegreen-labs/LUCAS_landscape_elements: LUCAS landscape elements ML worked example (zenodo.org) Instructions for accessing these data: data can be bulk downloaded from the provided URL or accessed through a STAC browser or any compatible library in data processing software.
Related research article	Martinez-Sanchez, Laura, Linda See, Momchil Yordanov, Astrid Verhegghen, Neija Elvekjaer, Davide Muraro, Raphaël d'Andrimont, Marijn van der Velde. “Automatic classification of land cover from LUCAS in-situ landscape photos using semantic segmentation and a Random Forest model.” Environmental Modelling & Software (January 2024). https://doi.org/10.1016/j.envsoft.2023.105931

## 1 Value of the Data

• Our dataset provides training data for machine learning segmentation tasks of street level landscape objects (cover, use, features), outside the context of urban navigation or automated driving.
• These landscape objects can provide critical context for land-use and land-cover assessments and comparison to satellite remote sensing data.
• In addition, segmented labels can be used to assess critical landscape and biodiversity related metrics, in the perspective of automating detection of e.g. woody features in the landscape, through citizen science or rapid survey efforts in support of (EU) biodiversity policies.

## 2 Background

The European Commissionʼs Land Use and Coverage Area frame Survey (LUCAS) data set is a crucial resource for land-use and land-cover statistics and monitoring performed every three years following a rigorous survey protocol to support various territorial policies [1]. This dataset provides harmonized and unbiased estimates of land-use and land-cover changes across the EU 27 and includes geo-referenced street view images (SVI) of every sample point.

At the same time, the improvements on the application of machine learning has increased the accuracy of products related to land cover (LC) and rural areas. These advancements have led to more accurate LC maps [2], improved analysis of climatic factors affecting LC classifications [3], and the creation of detailed crop type maps [4].

The integration of LUCAS data with machine learning algorithms, however, offers new opportunities to enhance the accuracy of the creation of high resolution LC maps [5], [6], [7].

Moreover, the accessibility of SVI has become a new stream of information to analyze rural and forested landscapes, providing detailed visual information that complements traditional data sources. Projects such as Pl@ntNet uses machine learning for plant identification from street view images, illustrating the potential of combining technology with visual data for agricultural and botanical research [8,9]. Furthermore, these images are being used to classify crop phenology stages, expanding the applications of street-level imagery in agricultural studies [10].

Recent research has explored the potential of combining crowd-source platforms with LUCAS SVI for crop identification using deep learning techniques [11], [12]. Going a step further by semantically segmenting LUCAS images has shown the possibility to classify LC classes and identify key image features for each LC type [13] and coupling with satellite data has help to bridge the gap between field survey data and satellite observations to enhance environmental monitoring and management practices in the future [14].

Semantic segmentation of agricultural SVI marks a significant advancement, offering a nuanced understanding of landscapes. This technique not only facilitates quick assessments of biodiversity metrics but also enables detailed analyses of landscape conditions, human impacts, and ecological health from a single image.

However, generating specific annotated data for machine learning applications remains a challenge. This process can be expensive, time-consuming, and sometimes ambiguous [8,9], leading to potential underuse of data due to access barriers or a lack of ancillary data. Making data accessible requires the data to be analysis-ready, through extensive curation for quality and amended with key ancillary data for research and development, for widespread use and re-use and implementation into policy workflows [15].

Yet, the applicability of this data within a machine learning and re-analysis context is key to support fast research developments, and the integration of this data into policy tools. Despite an increasing amount of available data, there is a continued need for more precise analysis ready data within the context of machine learning as all these applications rely on the availability of a validated and contextualized labeled dataset. Although analysis-ready data might not cover all use cases, domain knowledge ensures a wide range of applications.

As such, the LUCAS data as well as similar monitoring efforts [16] expand available training data of existing citizen science efforts and opens up the potential of the data to further re-analysis. Here, we provide an analysis-ready dataset of segmented LUCAS survey SVI, in support of land-use and land-cover change assessments, comparison and integration with Earth Observation based products, improved structural characterization of vegetation, as well as biodiversity and landscape heterogeneity monitoring. Additionally, evolutions of this dataset can be used in future applications in areas such as climate change impact assessment, sustainable agriculture planning, and agricultural development studies.

## 3 Data Description

**Fig. 1:**
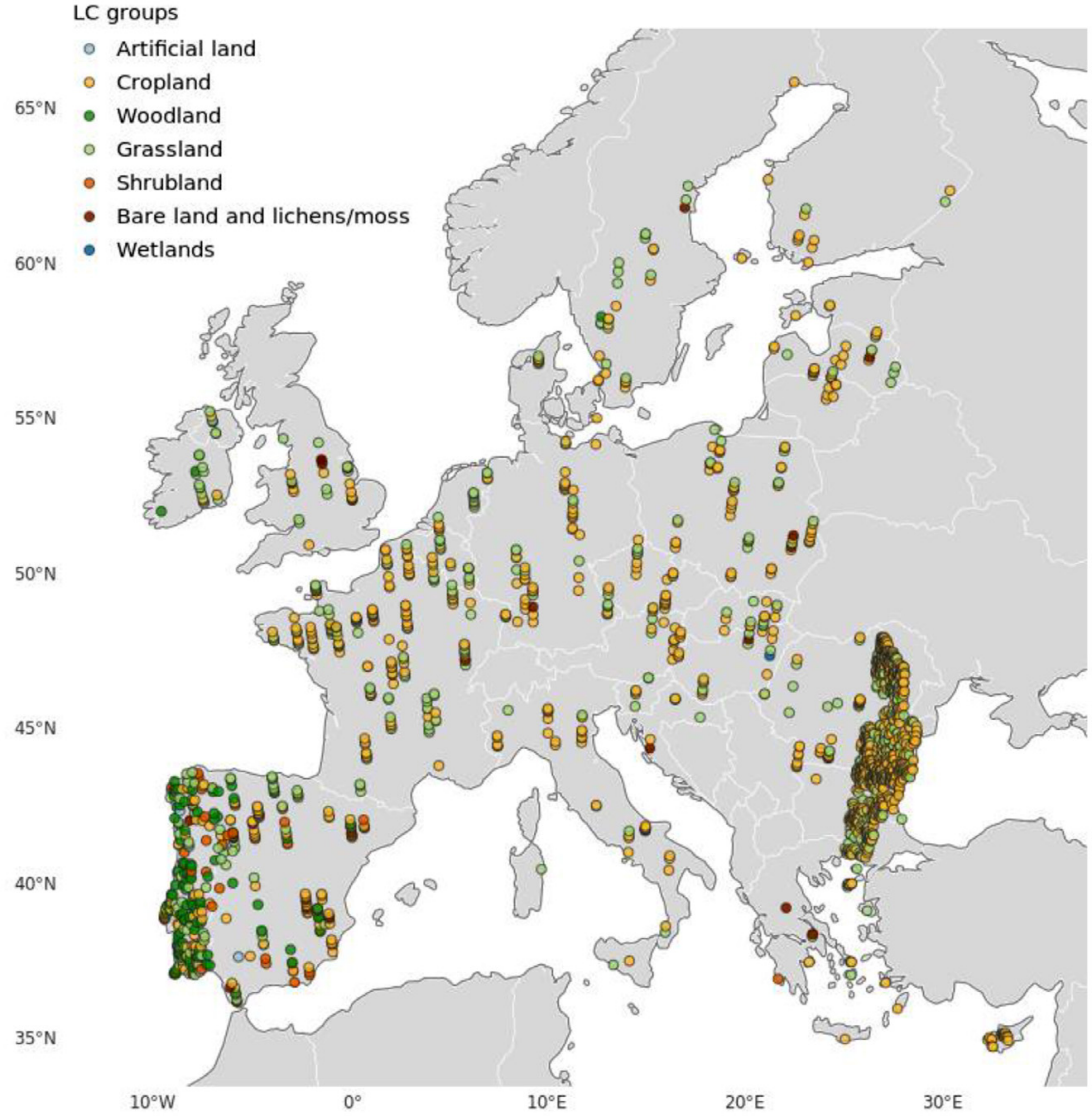
Overview map of the segmented LUCAS images labeled by land cover (LC) classes.

The segmented landscape SVI of 1784 north-looking landscape images were selected across Europe, but with high densities in Portugal, Bulgaria, Moldova and Romania (Fig. 1). As the LUCAS initiative originated within the context of agricultural statistics the sites are dominated by locations in managed landscapes, which is reflected in the representation of segmentation classes (see Table 2).

**Fig. 2:**
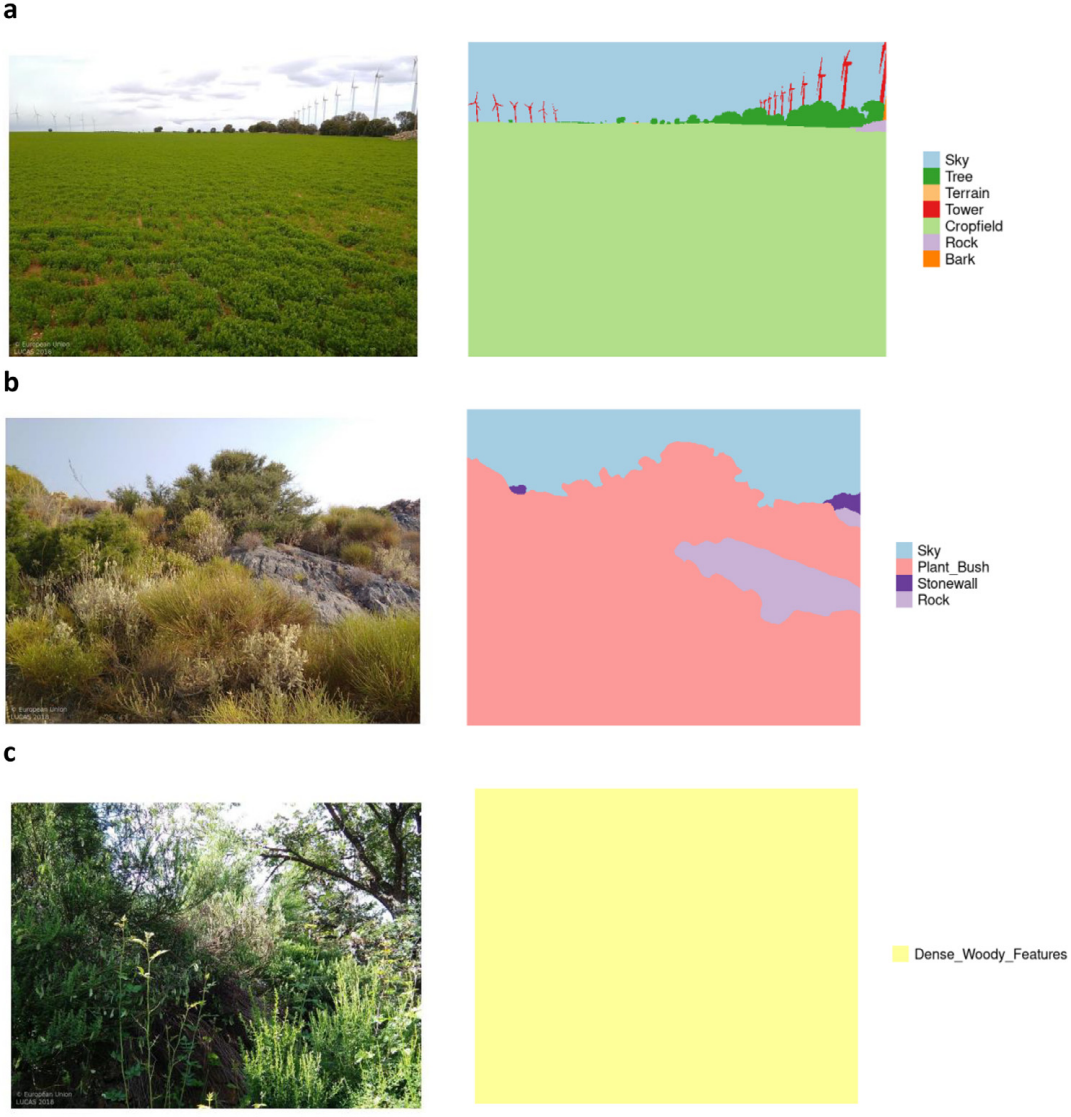
Image segmentation examples showing various landscapes (left panels) and their segmentation classes (right panel).

Selected images were segmented into 35 classes with data being presented as image and mask pairs (Fig. 2). Images are provided as RGB JPEG files, while masks are provided as single layer grayscale PNG files (with pixel values between 0 and 35). Segmentation classes were homogenized to their common denominator of which a total 35 classes were retained (Table 1).

**Table 1:** Segmentation classes and their descriptions, as used in the segmentation of the selected LUCAS images.

Class	Description
**Classes of importance**	
Tree	In this class we include individual trees, lines of trees and groups of trees, such as small forests in the distance. Some pictures include trees directly in view, some are far away and barely visible. We do not make a distinction between trees that are used for harvesting such as olive trees and others that are found in nature. Look at the class “Orchard”.
Orchard	We use this class to segment orchards with a visible tree pattern. There are two attributes for this class – near and far. Some we can see on distant hilltops, in which case we put them under “orchard - far”. Others are closer in the frame and we note them under “orchard - near”. The distance is of importance in this case because it varies the pattern that we see. Usually the ones further in the distance have a clearer planting pattern that could be useful and more recognizable for our computer vision. Note that if the picture is taken from within the orchard, and we can't see it in its entirety, then we would segment the individual trees and put them under the category “Tree” instead.
Dense_Woody_Features	We use this class to segment noisy pictures, usually taken from inside a forest where you can't see any individual trees, clear tree lines or clear tree crowns in their entirety. The images that include this class are usually a messy combination of branches, bark, sticks and leaves, very close in the frame.
Grass	We put all grasslands, both natural and artificially planted, in this class. Smaller patches of grass and grass in crop fields are included. Also dry grass, regardless of the color and texture. Minimal noise levels preferred. If the grass is mixed with too much soil, plants or rocks, we would put it under “Terrain”.
Field_Margin	Under this class we put grass margins, plant margins, flower margins and buffer strips. They are classified under “Field_Margin’’ with one of the four listed types added as an attribute (Buffer strip, Grass margin, Plant margin, Flower margin). Field margins are tricky, because they are very heterogeneous. We try our best to segment all of them but when in doubt we just use one of the other corresponding classes.
Earth_Ground	In this class we put pure soil, sand and crop fields that are in seeding or mulching stage. We use a checkbox attribute to indicate if the soil is a crop field or not.
Terrain	Under “Terrain” we put SVI that have noisy (mixed) terrain such as forest floors with leaves or patches of land that should not be classified as grasslands or any other category. Terrain is usually a mix between grass, small plants, weeds, leaves, soil, rocks etc.
Mountain	We use this to classify mountains and hills in the background. Usually far in the frame of the SVI. Mountains can also contain trees, villages, crop fields etc., but are too far away to segment separately.
Plant_Bush	Under this class we put everything that can be considered a plant, regardless of its shape and texture. Including bushes, hedges, weeds, cacti, etc. Excluding bushes with flowers in them. Those go in “Flower;Field”.
Cropfield	This includes all crop fields, regardless of what the crop is. They vary in size, texture, colour, shape, distance, etc. Crop fields that are in a seeding or mulching stage go to Earth;Ground.
Crop	We use this class for close up pictures of crops, where we can not see the field, because it is hidden behind an individual crop. In other cases we would see the field itself as well, but also a big clear crop that we could also delineate on top of it. Classes may overlap.
Rock	All natural rock formations. Usually medium and big. If they are miniature rocks we would not delineate them.
Waterbody	Water-bodies are scarce in the data set, so we make sure to delineate them perfectly every time. Further we have a choice of four types in the form of attributes. Those four are: sea, river, pond, lake.
Flowerfield	Under this class we put everything that has a high density of flowers, regardless of its nature and function. It could be a grassland with flowers, a tulip field, a rose bush, or just a small patch of the image with some flowers on it.
Animal	All animals go under this category.
Stonewall	Stonewalls.
**Other classes**	Classes that were used to segment objects that are not considered important for the project. The reason for that being that in order for the computer vision to work we had to segment 100% of the image. Sky, background, Building, Poles, Tower, Automobile, Path, Flower, Road, Wall, Bridge, Rail Transport, Traffic Sign, Lucas Marker, Bark, Fruit, Person, Well, Terrace

### 3.1 Dataset structure

The data are provided in a variety of data structures, both static and dynamic. The dynamic Spatio-Temporal Assets Catalog (STAC) facilitates the spatio-temporal subsetting of the data within the context of spatially explicit analysis such as comparisons with collocated remote sensing data. The dynamic utilization of the data allows for selective access without the need to download the entire dataset, especially as it continues to expand in size. This can be accomplished using the implementation of a Spatio-Temporal Assets Catalog, commonly used within the context of remote sensing imagery.

We also provide the data as a static dataset of images and masks facilitating conventional machine learning configurations. Both file structures provide access to the analysis-ready data and provide a quick way to test novel machine learning algorithms. Additional provided meta-data includes the S2GLC land-cover type [17], the geographic location of the LUCAS location, the view direction and homogenized image segmentation masks, and image class diversity metrics (see methods below). For reference only, the original dataset from which both analysis ready datasets are derived is provided.

#### 3.1.1 STAC (dynamic dataset)

The STAC format allows for easy spatio-temporal subsetting. Furthermore, through the STAC structure the dataset can be visually browsed using a STAC browser (https://radiantearth.github.io/stac-browser/#/external/data.source.coop/jrc-lucas/jrc-lucas-ml/stac/catalog.json). The STAC catalog can be queried using dedicated python and R packages, for example the pySTAC (https://pystac.readthedocs.io/) and the R {rstac} packages [18]. Ancillary data associated with the formal LUCAS database (19), in addition to the mask data, is embedded within the catalog itself. For these ancillary values we refer to the `lucas_ml_data.csv` dataset in the static dataset (below). The structure and specifications of a STAC catalog are outlined elsewhere (https://stacspec.org/).

#### 3.1.2 Static dataset

Together with the dynamic STAC data a standard machine learning dataset is provided with data divided into an `image` and `mask` directory in the main `ml_data` directory. The static data are consolidated and enhanced with the same ancillary data as provided in the STAC. This meta-data can provide the necessary context within machine learning exercises or exploratory analysis. Unlike the STAC catalog the data are provided separately as a file called `lucas_ml_data.csv` in the main `ml_data` directory. The main directory also contains the `classes_dataset.csv` CSV file containing the code and label correspondence used in harmonizing labels across the raw data (see Table 1). We provide a small worked example on how to train a segmentation algorithm using the static data [19].

#### 3.1.3 Raw data

For reference and transparency in processing we retained the raw data within this data release, in the `raw_data` folder. The original dataset is organized into batches, per segmentation campaign, with each batch containing three main folders, with `images` containing the LUCAS north-looking images captured for each theoretical point, the `full_masks` the pixel-level annotated masks as PNG files, a `partial_masks` folder for the first batch where some areas of the images are not delineated and finally a `classes_dataset.csv` CSV file containing the code and label correspondence in the main `raw_data` directory.

## 4 Experimental Design, Materials and Methods

LUCAS data are generally stratified according to basic EU regions for the application of regional policies (NUTS 2). Surveys are executed every three years starting in 2006, with the exception of the latest survey done in 2022, collecting land cover variables as well as SVI. More than 300,000 points were sampled during each survey, providing a statistical relevant sample across the domain. Across five campaigns, more than 5.4 million LUCAS survey SVI were collected, including so-called point and landscape SVI taken in the four cardinal directions at each point. A detailed methodological description, as well as the harmonized data and SVI are published in d'Andrimont et al. [20].

From the total LUCAS SVI database 1784 north-looking landscape images were selected across Europe. As the creation of this dataset originated within the context of agricultural statistics the selected sites are dominated by locations in managed landscapes such as ‘Cropland’ (*n* = 1122) and ‘Grassland’ (*n* = 463), with smaller contributions of ‘Woodland’ (*n* = 112), ‘Shrubland’ (*n* = 47), ‘Bare land and lichens/moss’ (*n* = 32), ‘Artificial land’ (*n* = 6) and ‘Wetlands’ (*n* = 2).

Selected images were segmented into 35 classes using the CVAT software, a tool to facilitate image segmentation for machine learning purposes (https://github.com/opencv/cvat/). Segmentation masks were outlined as JSON vector files. Vector masks were converted into raster image masks (png) with the same size as the original (LUCAS) images, having a width and height of 1600 by 1200 pixels, respectively (Fig. 2). Originally, a set of 48 segmentation classes was used; over time, these classes were gradually homogenized to their common denominator (most consistent comparable class) ending up with a total of 35 classes (see Table 1). Segmented LUCAS images were matched with the harmonized LUCAS database [20], containing the full dataset associated with every particular point location, on a site-by-site level. The LUCAS land cover data are classified along four levels of hierarchy (categories, classes, subclasses and spec), each level providing more detailed information [20]. The classes identified in the segmented LUCAS images align with the information in the LUCAS land cover classes level, and data are presented at this level (see Table 2). The full hierarchy of each image is available in the dataset (see `classes_dataset.csv` files).

**Table 2:** LUCAS land cover hierarchy and various levels (group, class, subclass), as well as the number of SVI found in the segmented dataset corresponding to a particular level.

Group	Class	Subclass	Nr of images
**A00 - Artificial land**			**Group total = 6**
	A20 - Artificial non-built up areas		Class total = 6
		A21 - Non built-up area features	3
		A22 - Non built-up linear features	3
**B00 - Cropland**			**Group total = 1122**
	B10 - Cereals		Class total = 661
		B11 - Common wheat	358
		B12 - Durum wheat	32
		B13 - Barley	109
		B14 - Rye	18
		B15 - Oats	22
		B16 - Maize	115
		B18 - Triticale	3
		B19 - Other cereals	4
	B20 - Root crops		Class total = 23
		B21 - Potatoes	13
		B22 - Sugar beet	8
		B23 - Other root crops	2
	B30 - Non-permanent industrial crops		Class total = 240
		B31 - Sunflower	122
		B32 - Rape and turnip rape	85
		B33 - Soya	14
		B34 - Cotton	9
		B35 - Other fibre and oleaginous crops	4
		B36 - Tobacco	1
		B37 - Other non-permanent industrial crops	5
	B40 - Dry pulses, vegetables and flowers		Class total = 30
		B41 - Dry pulses	19
		B43 - Other fresh vegetables	10
		B44 - Floriculture and ornamental plants	1
	B50 - Fodder crops		Class total = 137
		B51 - Clovers	5
		B52 - Lucerne	61
		B53 - Other Leguminous and mixtures for fodder	15
		B54 - Mixed cereals for fodder	12
		B55 - Temporary grasslands	44
	B70 - Permanent crops: fruit trees		Class total = 12
		B71 - Apple fruit	1
		B74 - Nuts trees	5
		B75 - Other fruit trees and berries	5
		B76 - Oranges	1
	B80 - Other permanent crops		Class total = 19
		B81 - Olive groves	11
		B82 - Vineyards	4
		B83 - Nurseries	1
		B84 - Permanent industrial crops	3
**C00 - Woodland**			**Group total = 112**
	C10 - Broadleaved woodland		Class total = 87
	C20 - Coniferous woodland		Class total = 20
		C22 - Pine dominated coniferous woodland	19
		C23 - Other coniferous woodland	1
	C30 - Mixed woodland		Class total = 5
		C32 - Pine dominated mixed woodland	3
		C33 - Other mixed woodland	2
**D00 - Shrubland**			**Group total = 47**
	D10 - Shrubland with sparse tree cover		Class total = 23
	D20 - Shrubland without tree cover		Class total = 24
**E00 - Grassland**			**Group total = 463**
	E10 - Grassland with sparse tree/shrub cover		Class total = 22
	E20 - Grassland without tree/shrub cover		Class total = 346
	E30 - Spontaneously re-vegetated surfaces		Class total = 95
**F00 - Bare land and lichens/moss**			**Group total = 32**
	F10 - Rocks and stones		Class total = 2
	F20 - Sand		Class total = 1
	F40 - Other bare soil		Class total = 29
**H00 - Wetlands**			Group total = 2
	H10 - Inland wetlands		Class total = 2
		H11 - Inland marshes	1
		H12 - Peatbogs	1

**Fig. 3:**
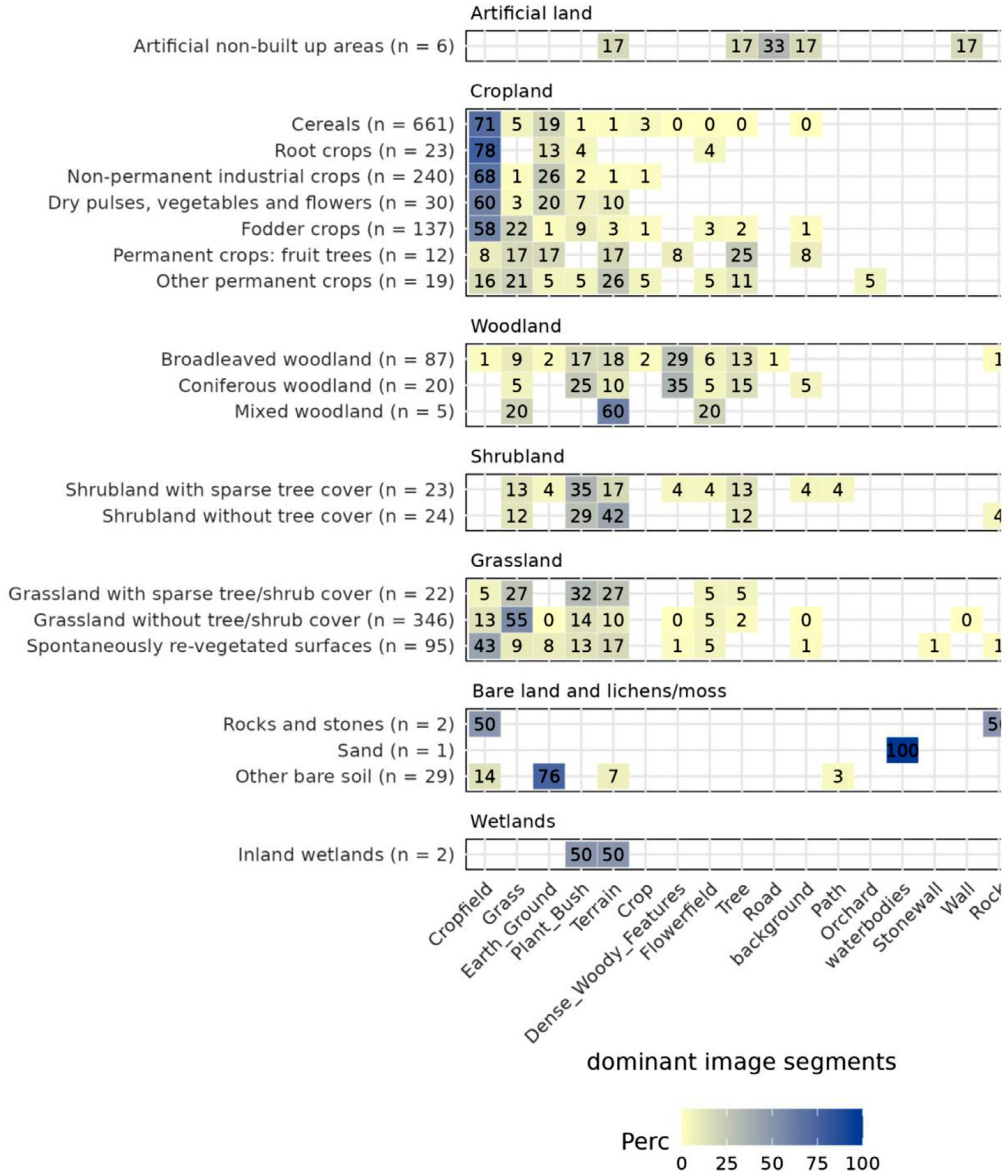
Cross comparison of LUCAS land cover (LC) classes microdata and dominant image segmentation labels. (Percentage = percentage of images in each class (total *n* = *x*) with the respective dominant LC subclass).

To compare the content of the segmented fractions with images with the overall land cover classes as contained within the LUCAS database we created cross tables across all available classes in both the database and the segmented image labels. In our analysis we focus only on land-based features, as such we ignored the fraction of the sky in our statistics. For each scene (image) we gathered the number of unique classes within the image, and the image label with the largest fractional coverage. We retained the majority class for each scene which was subsequently cross tabulated with the land cover label in the LUCAS database (Fig. 3).

## Limitations

In the compiled data set we bring together 1784 segmented images with existing and recently compiled LUCAS meta-data (19). Segmented labels covered 35 classes which are generally dominated by crop based labels (when excluding labeled sky pixels). This influence causes unbalanced data which needs to be addressed during machine learning tasks.

The dominant segments within the images correspond well with LUCAS land cover classes (Fig. 3). For example, croplands are generally dominated by ‘cropfield’ and ‘earth_ground’ segments, potentially related to the timing of image acquisition. Woodlands are dominated by ‘dense_woody_features’, ‘plant_bush’, ‘terrain’ and ‘trees’, and grasslands by ‘grass’, ‘plant_bush’ and ‘terrain’. We note that a holistic view needs to be considered when using any LUCAS data directly for in-situ validation of land-use and land-cover remote sensing data (for recent applications in this context see (5,6,7,13).

Some segmentations show interpretation issues. For example, 22% of fodder crop land cover images are dominated by ‘grass’, while the majority are classified as ‘cropfield’ (Figs. 2a, 3, Cropland panel). Grass in this instance could be classified as a crop. Other dominant segmentations might indicate a change in land-use over time. For example, 43% of spontaneously re-vegetated surfaces (grassland subclass) are dominated by ‘cropfield’ segments indicating farmers might have changed the land to use (Fig. 3, Grassland panel).

Few images are dominated by landscape artifacts related to the photographer's perspective, such as ‘rocks’, ‘roads’ and ‘stonewalls’ (Fig. 3, Bare land and lichens/moss panel). On average, the images contain 3.9 ± 1.8 segments (excluding sky), varying between a single segmentation found in 90 images of various land cover (Fig. 2c) and a maximum of 15 segments in an image of a fodder crop land cover.

The data are modest in size, but provide key data in the evaluation of biodiversity and land-use land-cover applications and have been key in recent validation and calibration exercises (5,6,7,13). The dataset as listed at https://source.coop/ is dynamic and future segmentation efforts will lead to increasing the dataset well over the current size. We invite the scientific community to grow this dataset where possible.

## Ethics Statement

The authors have read and follow the ethical guidelines put forward by Data in Brief. This work does not involve human subjects, animal experiments, or any data collected from social media platforms.

## CRediT Author Statement

**Laura Sanchez Martinez:** Methodology, Data-curation, preparation, writing- reviewing and editing. **Koen Hufkens:** Data curation, original draft preparation. **Elizabeth Kearsley:** Data curation, original draft preparation. **Dimitar Naydenov** Created the semantic segmentation. **Bálint Czúcz**: Methodology. **Marijn van der Velde:** Writing- reviewing and editing.

## Data Availability

Agri-environmental Semantic Segmentation of LUCAS landscape photos (Original data) (Source Cooperative)
